# Country-specific differences in the association between obesity risk factors in two high-income countries: a cross-sectional analysis from the Prospective Urban Rural Epidemiology (PURE) study

**DOI:** 10.1186/s12889-026-27980-3

**Published:** 2026-06-02

**Authors:** Christina E Lundberg, Martin Adiels, Victoria Miller, Paul Poirier, Sumathy Rangarajan, Martin Lindgren, Jon Edqvist, Hassan Mir, Koon K Teo, Philip Joseph, Scott A. Lear, Darryl P Leong, Salim Yusuf, Gilles R. Dagenais, Annika Rosengren

**Affiliations:** 1 Department of Food and Nutrition and Sport Science, Faculty of Education, University of Gothenburg, Gothenburg, Sweden, and Department of Molecular and Clinical Medicine, Institute of Medicine, Sahlgrenska Academy, University of Gothenburg, Gothenburg, Sweden, Läroverksgatan 5, Gothenburg, SE-405 30 Sweden; 2 School of Public Health and Community Medicine, Institute of Medicine, Sahlgrenska Academy, University of Gothenburg, Gothenburg, Sweden; 3 Population Health Research Institute, McMaster University and Hamilton Health Sciences, Hamilton, Ontario, Canada and Department of Medicine, McMaster University, Hamilton, Ontario Canada; 4 Faculté de pharmacie, Université Laval, and Institut universitaire de cardiologie et de pneumologie de Québec, Québec City, Canada; 5 Population Health Research Institute, McMaster University, Hamilton, ON Canada; 6 Department of Molecular and Clinical Medicine, Institute of Medicine, Sahlgrenska Academy, University of Gothenburg, Gothenburg, Sweden; 7 Department of Medicine Geriatrics and Emergency Medicine, Sahlgrenska University Hospital, Östra Hospital, Region Västra Götaland, Gothenburg, Sweden; 8 Division of Cardiology, University of Ottawa Heart Institute, University of Ottawa, Ottawa, Canada; 9 Population Health Research Institute, McMaster University and Hamilton Health Sciences, Hamilton, Hamilton, ON Canada; 10 Faculty of Healthy Sciences, Simon Fraser University, Burnaby, Canada and Centre for Heart Lung Innovation, Vancouver, Canada; 11 Quebec Heart and Lung Institute, Clinical Research Center, Laval University, Québec City, Qc Canada

**Keywords:** Obesity, Risk factors, Dietary intake, PURE study, Socioeconomic status

## Abstract

**Background:**

Although Sweden and Canada are high-income countries with similar social structures, obesity prevalence is significantly higher in Canada. This study explored country-specific differences in the association between socioeconomic and behavioural risk factors for obesity in Sweden and Canada, using data from the Prospective Urban Rural Epidemiological (PURE) study.

**Methods:**

This cross-sectional study included 9 790 adults aged 34–60 years from Canada (*n* = 6 652, 55% women) and Sweden (*n* = 3 138, 54% women). The Boruta algorithm was used to identify relevant factors that were associated with obesity among individual with normal weight (body mass index [BMI]: 18.5-<25 kg/m^2^) compared to individuals with obesity (BMI > 30 kg/m^2^). Logistic regression models with 95% confidence intervals (CI) estimated odds ratios (OR) of obesity by country in relation to selected risk factors for obesity.

**Results:**

More Canadians than Swedes had obesity (26 vs. 16%, p-value: <0.001). Ultra-processed food (UPF) and the Alternative Healthy Eating Index (AHEI-2010) score were identified as the main drivers of obesity risk. The highest UPF intake group was strongly associated with obesity in both Canada (OR = 2.31 [CI = 1.57–3.37]) and Sweden (OR = 2.83 [CI = 2.30–3.49]). Canadian men had higher UPF intake and were found to have a significantly higher risk of obesity (p for interaction: 0.032 and 0.021 for middle and highest tertiles), compared to Swedish males. Among women, low socioeconomic status (rural residence (OR = 2.30 [CI = 1.66–3.17] vs. OR = 1.42 [CI = 1.19–1.70]), low income (OR = 4.75 [CI = 2.71–8.95] vs. OR = 2.62 [CI = 2.16–3.18]), not working (OR = 2.24 [CI = 1.51–3.29] vs. OR = 1.32 [CI = 1.07–1.63]), and unskilled occupation (OR = 5.08 [CI = 3.05–8.45] vs. OR = 1.78 [CI = 1.38–2.28]) was more strongly associated with obesity in Sweden than in Canada.

**Conclusions:**

This study highlights important differences in obesity-related risk factors between Sweden and Canada. UPF consumption, the AHEI-2010 score and socioeconomic disadvantage emerged as key drivers of obesity, with notable sex- and country-specific patterns. These findings underscore the importance of tailored, context-specific public health strategies to address obesity in different national settings.

**Supplementary Information:**

The online version contains supplementary material available at 10.1186/s12889-026-27980-3.

## Background

The continuous increase in the prevalence of overweight and obesity is a significant public health challenge, and is associated with increased risk for cardiovascular disease, diabetes, and some cancers [1–3]. A recent study found that over 65% of all deaths in high-income countries can be attributed to a cluster of 12 modifiable cardiovascular risk factors, indicating that a vast majority of early deaths are preventable [4], with obesity being a major contributing risk factor.

Obesity results from a complex interaction between several factors. Established risk factors for developing obesity include demographic and socio-economic [5–8] (e.g., sex, age, education, income, marital status, and urban areas); mental health (e.g., depression); lifestyle factors [9, 10] (e.g., dietary, including alcohol, psychosocial, low levels of physical activity, grip strength, and sedentary behaviours); selected medical conditions [11] (e.g., asthma) and genetic factors [12]. On a societal level, the built environment has globally become increasingly obesogenic resulting from high availability of cheap ultra-processed energy-dense, often low in nutrients foods, and energy-rich drinks [13], promoting physical inactivity and sedentary behaviours in occupational and leisure settings, and alterations in sleep patterns [14, 15]. Today, ultra-processed foods (UPFs) constitute a dominant segment of the global food system and often account for a large share of the food supply in high-income countries [13]. UPFs are typically designed to be hyper-palatable, attractive, convenient, and shelf-stable, which may promote overconsumption [16]. These transformations in food systems and diet quality, particularly the rising intake of UPFs, have contributed to the global burden of malnutrition in all its forms, including overnutrition, and have been associated with elevated body mass index (BMI), insulin resistance, inflammation, and high blood pressure [17]. For the individuals susceptible to obesity, an obesogenic environment results in the accumulation of fat in the fat cells leading to an increasing number of fat cells and, over time greater difficulty maintaining a normal body weight [18].

Although rates of overweight and obesity are increasing world-wide, across countries at all income levels, there are vast differences between seemingly similar countries [1, 19]. For instance, Sweden and Canada are two high-income countries (HIC) with many similarities such as population composition, wealth, social welfare, and healthcare systems, but a marked difference in obesity rates, with recent estimates in Swedish women and men of 24.3% and 18.3%, respectively, and corresponding estimates for Canadian women and men 28.5% and 24.9%, respectively [1]. Data from the worldwide Prospective Urban Rural Epidemiological (PURE) study [20] provide a unique opportunity to compare the associations of potential causes of obesity in two high-income countries using the same robust methodology. In doing so, we identify key national features that could help form context-specific strategies for prevention. Therefore, the aim of the present study was to identify country specific differences in the association between risk factors for obesity in Sweden and Canada.

## Methods

### Study design and participants

This was a cross-sectional analysis of PURE participants from Canada and Sweden aged 34 to 60 years from the PURE study [20]. We only included individuals aged 60 years and younger, due to the associations between weight loss and morbidity that is becoming increasingly evident in older people [21]. In the final study sample, we excluded individuals with no information on dietary factors (*n* = 547), implausible energy intake *≥* 5 000 kcal (*n* = 125), height < 140 cm (*n* = 13), BMI > 60 kg/m^2^ (*n* = 31), implausible height or weight (*n* = 6), and underweight (*n* = 73) (SFigure 1).

The PURE study was designed to include countries across a broad range of economic levels, social circumstances, and health policies, with a proportionally larger representation from low-income and middle-income countries (LICs and MICs) [4, 22]. The study included four HICs: Canada, Saudi Arabia, Sweden and the United Arab Emirates. Because Sweden and Canada share several characteristics, related to population composition, wealth, social welfare, and healthcare systems [23, 24], participants from these two countries were selected for this study. In all participating countries, urban and rural communities were selected using pre-specified criteria (Supplementary Appendix A). Within each community, households and individuals were selected using sampling strategies designed to minimize selection bias of individuals who could potentially introduce bias into the associations between risk factors and outcomes [4, 22].

## Procedures and risk factors

We evaluated how the risk of obesity was associated with 23 selected risk factors, available in the PURE study, that are supported in the literature as possible individual-level causal risk factors for the development of obesity [5–12]. The aim was to include potential upstream risk factors rather than conditions more commonly considered consequences of obesity and therefore more likely to lie on the causal pathway. The primary outcome was the odds of having obesity at baseline, defined as a BMI *≥* 30 kg/m^2^. The risk factors used in our analysis were obtained at the baseline examination which occurred between 2005 and 2009 in Sweden and 2006 to 2009 in Canada, and collected using standardised methods at the community, household, and individual levels [25].

A detailed summary of methods of measurement and the categorization for assessing the risk factor in relation to the outcome for each risk factor is available in the Supplementary Appendix B (STable 1). Socio-demographic risk factors were: sex, area of residence (rural or urban), ethnicity (European/ non-European), community socioeconomic status (SES) (high, middle and low), educational level (primary, secondary, trade/ collage/ university), occupation (professional, skilled, unskilled), working status (yes/ no), household income (high, middle and low), and proportion of household income spent on food per month (high, middle and low).

Self-reported behavioural factors include tobacco use (current and never/ former), heavy alcohol consumption (yes/ no), global stress (yes/ no), and sleep duration (6–9 h/ <6 or > 9 h). Physical activity was measured using the International Physical Activity Questionnaire (IPAQ) and classified as meeting physical activity guidelines as defined by world health organization (WHO) of at least 150–300 min of moderate-intensity or 75–150 min of vigorous-intensity aerobic activity weekly (yes/ no). Grip strength was measured using JAMAR dynamometer in kg with the dominant hand (high, middle and low), and sitting time (high, middle and low).

Dietary intake was measured using country-specific validated food frequency dietary questionnaires (FFQ) [26, 27]. Country specific food composition tables were used to estimate daily energy and nutrient intake. In the present study, ultra-processed food (UPF) was classified based on the NOVA classification [28]. UPF was defined as industrially nutrient-poor, and energy-dense products containing minimal whole foods and various additives to enhance taste, texture, and shelf life [29]. The full definition of UPF in PURE have previously been described [30]. Overall dietary quality was assessed by an adapted version of the Alternative Healthy Eating Index-2010 (AHEI-2010) score. The AHEI-2010 score is based on 11 components: vegetables, fruit, whole grains, nuts and legumes, omega–3 fatty acids, polyunsaturated fatty acids (PUFA), sugar-sweetened beverages (SSB) and fruit juice, alcohol, unprocessed red meat and processed meat, trans-fats, and sodium [31]. However, we were unable to estimate trans-fat intake in the PURE study. Therefore, a modified score based on 10 components was used, which does not include trans-fat. Each component scored from 0 to 10, with a maximum total score of 100, where higher scores indicate healthier diets. This score was further divided into quintiles. Additional included dietary factors were energy intake (high, middle and low), and UPF intake (high, middle and low).

Medical conditions included antidepressant medication use, as a proxy for depression/ mental health (yes/ no), and asthma (yes/no).

### Statistical analysis

Means and standard deviations (SD) for continuous variables and counts and percentages for categorical variables are presented in Table 1. Baseline data in Table 1 are reported without imputation. Missing data were imputed using Multivariate Imputation by Chained Equations (MICE) with five imputed datasets [32]. MICE perform multiple imputations by iteratively modelling each incomplete variable as a function of the others in a series of regression models. The five generated imputed datasets each represents a plausible set of values for the missing data. Analyses were conducted separately on each dataset, and results were subsequently pooled using Rubin’s rules to obtain estimates that reflect both within- and between-imputation variability [32]. The information on missing values for included variables is shown in STable 2. Variables included in the MICE algorithm were sex, area of residence, ethnicity, community SES, social status, educational occupation, working status, household income, percent of household income spent on food, tobacco status, physical activity, grip strength, alcohol consumption, stress, sitting time, sleep duration, AHEI-score, ultra-processed food, energy intake, and antidepressant medication (STable 1). Statistical comparisons were performed using chi-square ($\:{x}^{2}$) test for categorical variables and the Wilcoxon rank-sum test for medians to assess differences in risk factors between countries.

**Table 1:** Baseline characteristics of cohort, without imputation

	Overall (*n* = 9 790)	Sweden (*n* = 3 138)	Canada (*n* = 6 652)	*p*-value
Anthropometrics				
Weight, kg	78.7 (17.0)	78.2 (14.8)	78.9 (17.9)	0.093
Height, cm	169.9 (9.5)	172.2 (9.4)	168.9 (9.4)	< 0.001
BMI (kg/m^2)^	27.2 (5.1)	26.3 (4.2)	27.6 (5.4)	< 0.001
High waist/hip ratio, n (%) *	2 656 (44.8)	733 (39.3)	1 923 (47.3)	< 0.001
High waist circumference**	1 925 (32.4)	446 (23.9)	1 478 (36.3)	< 0.001
BMI group				< 0.001
Normal weight (BMI = 18.5-<25)	3 718 38.0)	1 356 (43.2)	2 362 (35.5)	
Overweight (BMI = 25.0-<30)	3 856 (39.4)	1 272 (40.5)	2 584 (3.8)	
Obesity (BMI = ≥ 30)	2 216 (22.6)	510 (16.3)	1 706 (25.6)	
Demographic factors				
Sex, men, n (%)	4 445 (45.4)	1 449 (46.2)	2 996 (45.0)	0.302
Age, years	49.3 (6.8)	49.1 (7.0)	49.4 (6.7)	0.086
Area of residence, n (%)				
* Rural*	2 657 (27.1)	597 (19.0)	2 060 (31.0)	< 0.001
Ethnicity, n (%)				
* European*	8 985 (91.8)	2 951 (94.0)	6 034 (90.7)	< 0.001
Community SES, n (%)				< 0.001
* Low*	2 171 (22.2)	471 (15.0)	1 700 (25.6)	
* Middle*	4 919 (50.2)	1 889 (60.2)	3 030 (45.6)	
* High*	2 700 (27.0)	778 (24.8)	1 922 (28.9)	
Socioeconomic factors				
M*arried/ living partner*, n (%)	7 831 (80.0)	2 522 (80.4)	5 309 (79.8)	0.587
Education, n (%)				< 0.001
* Primary or less*	501 (5.1)	360 (11.5)	141 (2.1)	
* Secondary*	2 918 (29.8)	1139 (36.3)	1 779 (26.7)	
* Trade*,* collage/ university*	6 364 (65.0)	1 638 (52.2)	4 726 (71.0)	
Occupation, n (%)				< 0.001
* Professional*	5 048 (51.6)	1 546 (49.3)	3 502 (52.6)	
* Skilled*	3 753 (38.3)	1 394 (44.4)	2 360 (35.5)	
* Unskilled*	961 (9.8)	196 (6.2)	765 (11.5)	
Not working, n (%)	1 322 (13.5)	300 (9.6)	1 022 (15.4)	< 0.001
Household income, mean (SD)	5 269.6 (2346.7)	5 155.9 (2 850.1)	5 328.4 (2 035.8)	0.001
Household income, % spent on food/month, mean (SD)	11.0 (6.4)	11.24 (5.5)	10.9 (6.7)	0.042
Behavioural factors				
Tobacco use, n (%)	1 698 (17.3)	769 (25.4)	902 (13.6)	< 0.001
Heavy alcohol consumption, n (%)	2 530 (25.8)	576 (18.4)	1 954 (29.4)	< 0.001
Global stress, n (%)	4 520 (46.2)	1 516 (48.3)	3 004 (45.2)	0.004
Sleep duration, 6–9 h, n (%)	7 473 (76.3)	2 584 (82.3)	4 889 (73.5)	< 0.001
Meets physical activity recommendations, n (%)	8 272 (84.5)	2 746 (87.5)	5 526 (83.1)	< 0.001
Grip strength (kg), dh	37.7 (12.7)	39.8 (12.6)	36.6 (12.6)	< 0.001
Sitting time, minutes, mean (SD)	280.4 (143.7)	259.8 (131.0)	290.2 (148.3)	< 0.001
Diet, mean (SD)				
Energy intake, kcal/ day	2 309.3 (833.1)	2 144.8 (748.6)	2 386.9 (859.4)	< 0.001
Ultra processed foods, g/ day	268.3 (253.1)	140.5 (154.5)	328.6 (267.8)	< 0.001
AHEI score, mean	56.4 (11.4)	55.2 (10.3)	56.9 (11.9)	< 0.001
AHEI components				
* Vegetables*,* g/ day*	439.0 (281.1)	355.4 (229.5)	478.4 (294.3)	< 0.001
* Fruits*,* g*	237.6 (175.3)	249.5 (194.7	232.0 (165.0)	0.001
* Whole grain*,* g*	117.3 (110.6)	96.7 (77.7)	127.0 (121.9)	< 0.001
* Nuts and legumes*,* g*	49.0 (44.8)	39.1 (45.2)	53.6 (43.9)	< 0.001
* OMEGA 3*,* g*	8.8 (2.2)	9.5 (1.4)	8.4 (2.4)	< 0.001
* PUFA*,* E%*	4.7 (1.2)	4.8 (1.1)	4.7 (1.3)	<0.001
* SSB and fruit juice*,* g*	209.4 (240.5)	126.5 (176.0)	155.0 (231.7)	< 0.001
* Alcohol*,* g*	138.3 (205.5)	102.9 (126.7)	154.9 (231.7)	< 0.001
* Unprocessed read meat* *and processed meat*,* g*	94.2 (59.3)	91.1 (48.0)	95.6 (63.9)	<0.001
* Sodium*,* mg*	3 147.4 (1 206.2)	3 415.6 (1 169.2)	3 020.9 (1 190.1)	< 0.001
Comorbidity				
Antidepressants, n (%)	947 (9.7)	225 (7.2)	722 (10.9)	< 0.001

*BMI* Body mass index, *Dh *Dominant hand, *kcal* kilocalorie, *AHEI* Alternative Healthy Eating Index, *g *gram, *mg *milligram, *OMEGA 3 * Omega–3 fatty acids, *PUFA* Polyunsaturated fatty acids, *E% *Percentage of energy, *SSB *Sugar-sweetened beverages

* High waist/ hip ratio: > 0.9 for men and > 0.85 for women

** High waist circumference: ≥102 cm for men and ≥ 88 cm for women

In further analyses, only individuals with normal weight (body mass index [BMI] 18.5 to < 25.0 kg/m²) or obesity (BMI ≥ 30.0 kg/m²) were included. Thus, individuals with underweight or overweight (*n* = 3,930) were excluded from the stratified analyses comparing normal weight and obesity. The Boruta package in R [33] was deployed to identify which of the 23 included risk factors that were deemed unimportant in relation to the outcome in the present study. The Boruta algorithm is built around the random forest classification model. This technique repetitively evaluates variable importance by comparing the provided variables with so called shadow variables, providing an evidence-based assessment of variable relevance. Variables that were not identified as important by the Boruta procedure were excluded from further analysis in this study. To assess whether overlap between the dietary variables influenced the Boruta results, we performed sensitivity analyses in which the model was rerun three times, each time including only one dietary variable at a time (AHEI-2010, ultra-processed food intake, or total energy intake). The analyses showed highly similar variable importance across analyses, suggesting that the identification of important risk factors was not driven by overlap between the dietary variables (SFigure 2–4).

Separate logistic regression models were used to estimate odds ratios (ORs) and 95% confidence intervals (CIs) for each risk factor associated with obesity. Contrast matrices were applied to compare ORs between countries. In Model 1, a multivariable model adjusted for age and sex, we tested odds of obesity for each risk factor by country. In Model 2, also adjusted for age and sex, country was included as an interaction term with the outcome, obesity. Differences in risk between countries are illustrated with p-values for interaction. All models that included dietary factors were additionally adjusted for total daily energy intake. P-values were adjusted for multiple hypothesis testing using the Holm-Bonferroni method [34]. A two-sided p-value < 0.05 was considered statistically significant. Statistical analyses were performed in R version 4.3.0 (The R Project for Statistical Computing, Vienna, Austria).

## Results

A total of 9 790 men and women aged 35 to 60 years from the PURE study were included from Canda (*n* = 6 652, 55.0% women) and Sweden (*n* = 3 138, 54.8% women. Baseline characteristics without imputation are summarised in Table 1. Canadians, compared with Swedish participants, had a higher mean BMI (27.6 vs. 26.3 kg/m^2^, *p*-value: <0.001), and a higher percent had obesity (25.6 vs. 16.3%, *p*-value: <0.001). More Canadians than Swedes lived in rural areas (31.0% vs. 19.0%, *p*-value: <0.001), in areas with lower SES (25.6% vs. 15.0%, *p*-value: <0.001), did not work 15.4% vs. 9.6%, *p*-value: <0.001), but had a higher proportion of individuals with a high education (71.0% vs. 52.2%, *p*-value: <0.001). The proportion of participants who were married/cohabiting, household income, and the percentage of income spent on food per month were similar across countries **(**Table 1**).**

Swedish participants were more likely to report that they slept 6–9 h per night (82.3 vs. 72.5%, *p*-value: <0.001), met physical activity recommendations (reported *≥* 150 min a week) (87.5 vs. 83.1%, *p*-value: <0.001), less sitting time (259.8 vs. 290.2 min/ day, p-value: <0.001), and less heavy alcohol consumption (18.4 vs. 29.4%, *p*-value: <0.001). In contrast, Canadian participants reported lower use of tobacco (13.6 vs. 25.4%, *p*-value: <0.001) and a greater adherence to the AHEI-2010 score (mean AHEI score of 56.9 and 55.2, *p*-value < 0.001 compared with Swedish participants. Canadians also reported an overall higher daily energy intake (2 389.9 vs. 2 144.9 kcal, *p*-value < 0.001) and a markedly higher intake of UPF (328.6 vs. 140.5 g, *p*-value < 0.001) (Table 1). Reported daily intakes in gram/ mg of each of the AHEI components for the total cohort is presented in Table 1 and by BMI category, only including those with normal weight and obesity, in Table 2. Baseline characteristics with imputation, stratified by BMI category is summarised in STable 3, and by BMI category and sex are summarised in STable 4.

**Table 2:** Baseline characteristics of cohort, by BMI category, without imputation

	Sweden (*n* = 1 866)		Canada (*n* = 4 068)		*p*-value
	Normal weight (*n* = 1 356)	Obesity (*n* = 510)	Normal weight (*n* = 2 362)	Obesity (*n* = 1 706)	
Anthropometrics					
Weight, kg	67.4 (8.8)	98.0 (13.3)	63.8 (8.7)	99.1 (16.4)	< 0.001
Height, cm	171.4 (9.1)	171.4 (9.7)	167.6 (8.9)	168.7 (9.7)	< 0.001
BMI (kg/m^2)^	22.9 (1.5)	33.3 (3.5)	22.6 (1.6)	34.8 (4.8)	< 0.001
High waist/hip ratio, n (%) *	325 (24.0)	408 (80.0)	650 (27.5)	1 273 (74.6)	< 0.001
High waist circumference**	13 (1.0)	433 (84.9)	33 (1.4)	1 446 (84.8)	< 0.001
Demographic factors					
Sex, men, n (%)	481 (35.5)	258 (50.6)	746 (31.6)	834 (48.9)	< 0.001
Age, years	48.6 (7.1)	49.6 (7.0)	48.4 (6.8)	50.1 (6.7)	< 0.001
Area of residence, n (%)					
* Rural*	223 (16.4)	136 (26.7)	634 (26.8)	612 (35.9)	< 0.001
Ethnicity, n (%)					
* European*	1 290 (95.1)	477 (93.5)	2 146 (90.9)	1 562 (91.6)	< 0.001
Community SES, n (%)					< 0.001
* Low*	170 (12.5)	95 (18.6)	558 (23.6)	486 (28.5)	
* Middle*	818 (60.3)	312 (61.2)	1 038 (43.9)	812 (47.6)	
* High*	368 (27.1)	103 (20.2)	766 (32.4)	408 (23.9)	
Socioeconomic factors					
M*arried/ living partner*, n (%)	1 078 (79.5)	410 (80.4)	1 886 (79.8)	1 330 (78.0)	0.441
Education, n (%)					< 0.001
* Primary or less*	123 (9.1)	90 (17.6)	28 (1.2)	57 (3.3)	
* Secondary*	454 (33.5)	213 (41.8)	519 (22.0)	579 (33.9)	
* Trade*,* collage/ university*	779 (57.4)	207 (40.6)	1 815 (76.8)	1 070 (62.7)	
Occupation, n (%)					< 0.001
* Professional*	720 (53.1)	195 (38.2)	1 383 (58.6)	775 (45.4)	
* Skilled*	561 (41.4)	265 (52.0)	717 (30.4)	697 (40.9)	
* Unskilled*	75 (5.5)	50 (9.8)	262 (11.1)	234 (13.7)	
Not working, n (%)	119 (8.8)	81 (15.9)	341 (14.4)	334 (19.6)	< 0.001
Household income, mean (SD)	5 239.9 (2 704.4)	4 811.4 (3 065.2)	5 569.7 (2 009.9)	4 987.3 (2 153.3)	< 0.001
Household income, % spent on food/month, mean (SD)	11.2 (5.9)	12.1 (6.3)	10.8 (6.5)	11.2 (7.0)	0.001
Behavioural factors					
Tobacco use, n (%)	323 (23.8)	119 (23.3)	294 (12.4)	251 (14.7)	< 0.001
Heavy alcohol consumption, n (%)	215 (15.9)	92 (18.0)	664 (28.1)	447 (26.2)	< 0.001
Global stress, n (%)	669 (49.3)	233 (45.7)	1 051 (44.5)	852 (49.9)	0.002
Sleep duration, 6–9 h, n (%)	1 140 (84.1)	401 (78.6)	1 743 (73.8)	1 208 (70.8)	< 0.001
Meets physical activity recommendations, n (%)	1 310 (96.6)	467 (91.6)	2 245 (95.0)	1 526 (89.4)	< 0.001
Grip strength (kg), dh	37.9 (11.5)	40.1 (13.8)	34.1 (11.2)	36.7 (13.5)	< 0.001
Sitting time, minutes, mean (SD)	255.5 (128.9)	274.0 (135.7)	284.3 (146.8)	299.1 (152.3)	< 0.001
Diet, mean (SD)					
Energy intake, kcal/ day	2 120.6 (737.8)	2 129.5 (776.7)	2 301.9 (821.9)	2 470.1 (896.8)	< 0.001
Ultra processed foods, g/ day	129.5 (129.7)	166.2 (211.5)	269.7 (214.0)	405.8 (319.1)	< 0.001
AHEI score, mean	56.4 (10.2)	52.6 (10.2)	60.0 (11.7)	53.3 (11.2)	< 0.001
AHEI components					
* Vegetables*,* g/ day*	366.6 (239.2)	343.5 (227.4)	486.4 (279.5)	473.0 (307.9)	< 0.001
* Fruits*,* g*	254.9 (189.2)	239.3 (199.0)	247.1 (164.1)	216.6 (164.0)	< 0.001
* Whole grain*,* g*	61.8 (55.5)	55.4 (57.1)	103.6 (107.0)	93.1 (97.2)	< 0.001
* Nuts and legumes*,* g*	40.8 (47.8)	36.1 (44.0)	55.9 (44.4)	50.7 (42.7)	< 0.001
* OMEGA 3*,* g*	9.5 (1.3)	9.4 (1.6)	8.5 (2.4)	8.4 (2.4)	< 0.001
* PUFA*,* E%*	4.8 (1.2)	4.8 (1.2)	4.7 (1.3)	4.8 (1.3)	0.006
* SSB and fruit juice*,* g*	123.2 (155.2)	135.6 (226.3)	201.3 (204.7)	307.3 (308.4)	< 0.001
* Alcohol*,* g*	99.6 (124.2)	93.1 (124.4)	142.4 (203.7)	142.3 (242.3)	< 0.001
* Unprocessed read meat* *and processed meat*,* g*	86.7 (46.6)	102.7 (56.2)	80.8 (57.8)	111.6 (65.9)	< 0.001
* Sodium*,* mg*	3 342.2 (1 190.6)	3 490.2 (1 283.5)	2 880.9 (1 118.5)	3 203.1 (1 243.3)	< 0.001
Comorbidity					
Antidepressants, n (%)	78 (5.8)	51 (10.0)	205 (8.7)	273 (16.0)	< 0.001

*SES* Socioeconomic status, *Dh *Dominant hand, *h *hours, *kcal* kilocalorie, *AHEI* Alternative Healthy Eating Index, *g *gram, *mg *milligram, omega–3 fatty acids, *PUFA* Polyunsaturated fatty acids, *SSB *Sugar-sweetened beverages

**Fig. 1:**
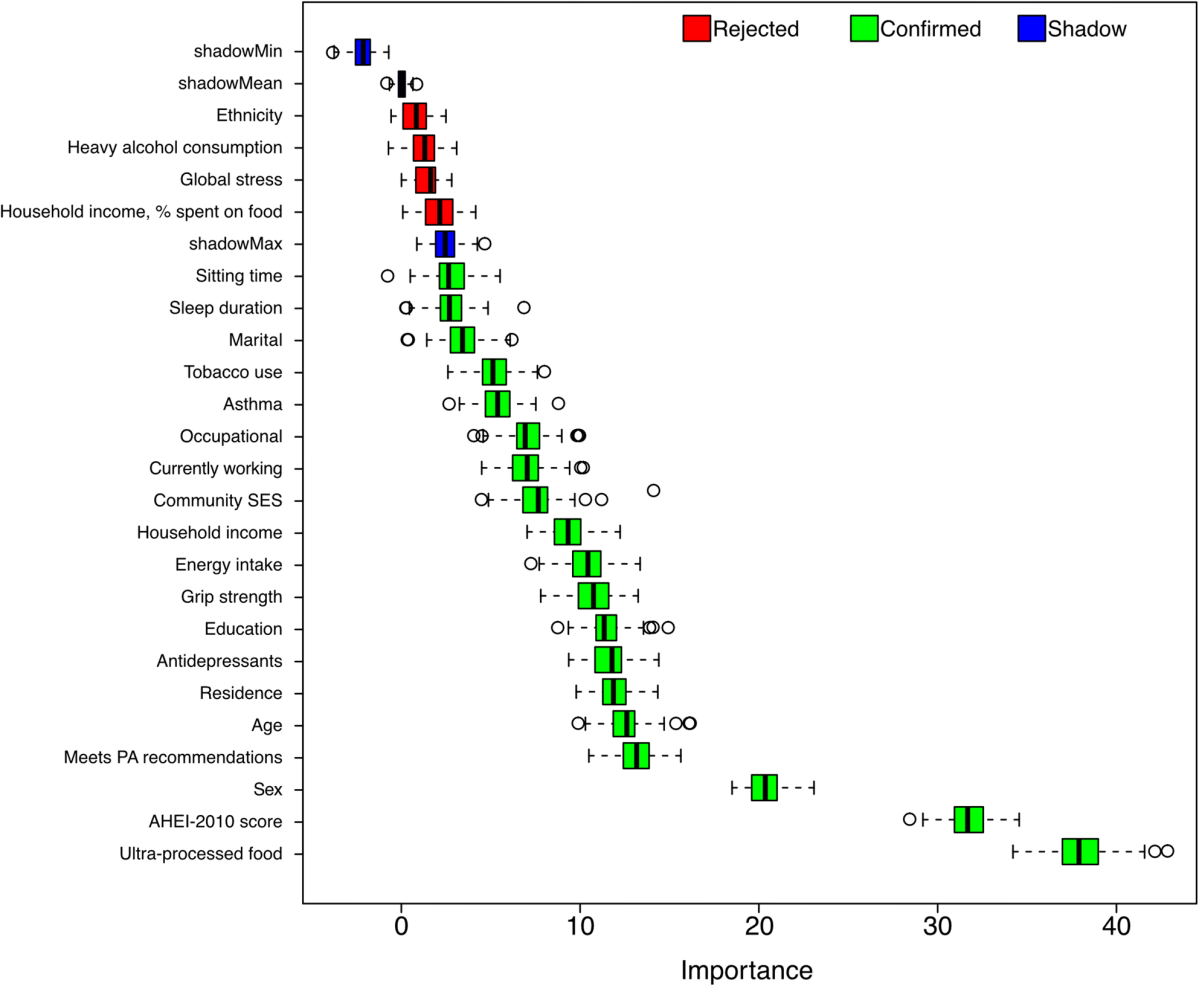
Variable importance of risk factors. Boxplot representation of variable importance as determined by the Boruta algorithm. Green boxplots indicate variables confirmed as important for having obesity. Red boxplots represent variables deemed unimportant and excluded from further analysis. Blue boxplots correspond to shadow variables, which serve as a reference to assess variable relevance. Variables with importance scores significantly higher than the shadow variables were retained for further analysis

The Boruta analysis identified most variables as important factors for having obesity. Among the 23 risk factors analysed using the Boruta algorithm, UPF and the AHEI-2010 score emerged as highly important factors of obesity, each with an importance score between 30 and 40. Additionally, sex showed moderate importance (scores around 20), while the remaining variables had importance scores ranging from around 5 to 10. The Boruta analysis classified global stress, heavy drinking, percent of income spent on food, and ethnicity as not important, indicating they did not contribute significantly to obesity in this cohort. These variables were excluded from further analysis. A summary of the variable importance rankings is presented in Fig. 1.

**Fig. 2:**
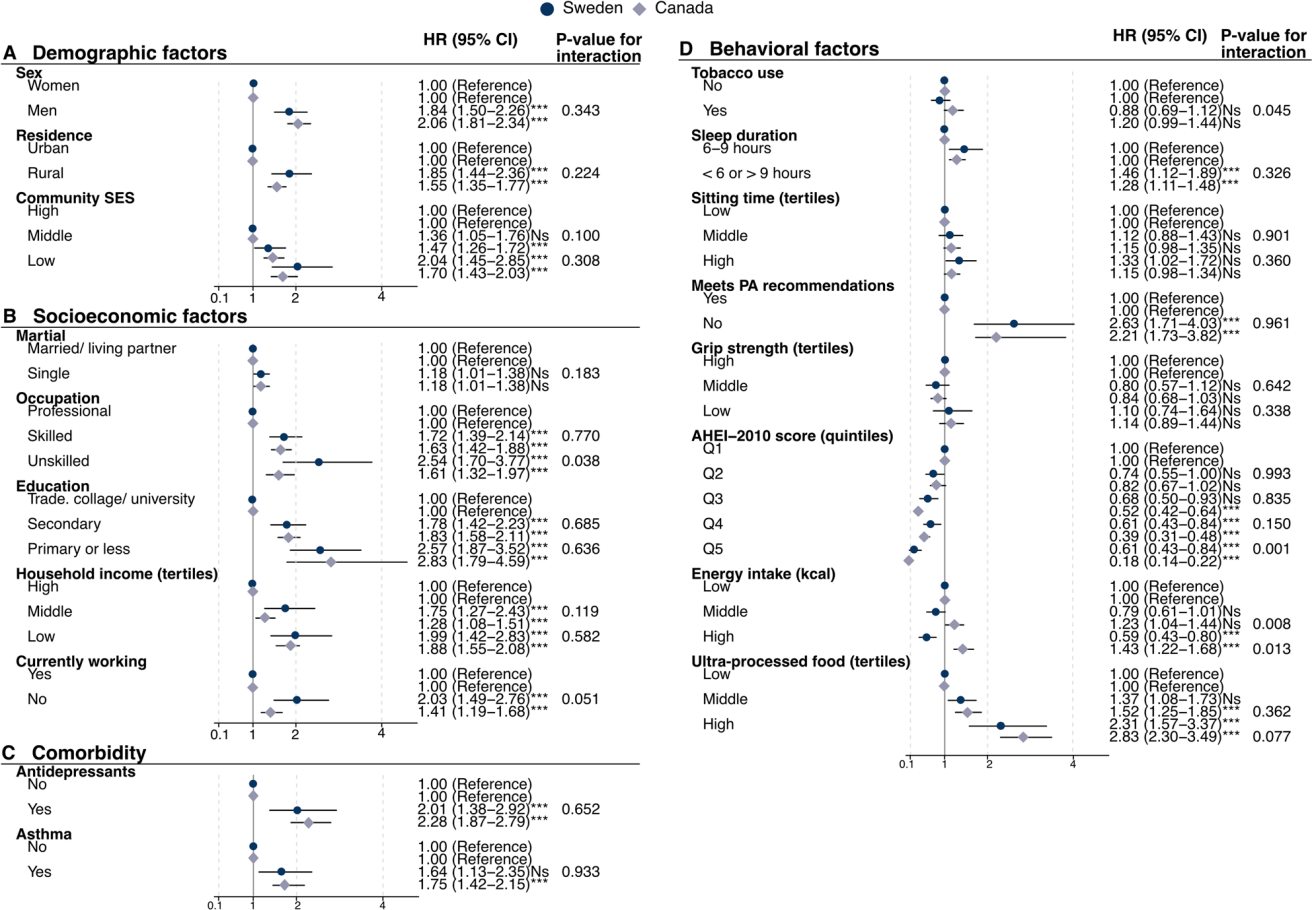
Association between socio-demographic, behavioural risk factors, and comorbidity and odds of obesity. * are used to indicate interaction between countries and the outcome. *= *p* value: <0.05, **= *p* value: <0.005, ***= *p* value: <0.001. SES= socioeconomic status. Q=quintile. AHEI= Alternative Healthy Eating Index. kcal= kilocalorie. g=gram

Figure 2 shows the association between (a) demographic, (b) socioeconomic, (c) comorbidity, and (d) behavioural risk factors for having obesity by country, with normal weight as a reference. Factors associated with obesity in both countries were male sex, living in a rural area or community with low SES, being a skilled or unskilled worker, having low level of education, having low or middle level of income, not working, being on anti-depressants, seeping less than 6 h or more than 9 h per day, not meeting physical activity recommendations, and a low AHEI-2010 scores **(**Fig. 2**)**. Odds of obesity increased linearly with higher intake of UPF and decreased linearly with higher AHEI-2010 scores in both countries. Canadians in AHEI-2010 quintile five had a significantly lower risk of obesity compared with Swedes in the same quintile (Canada: OR = 0.18, CI = 0.14–0.22; Sweden: OR = 0.61, CI = 0.43–0.84, p-value for interaction = 0.001) **(**Fig. 2**)**. There was a tendency for all indicators of lower socioeconomic status to be associated with a higher risk of obesity among Swedes compared to Canadians; however, only having an unskilled occupation reached statistical significance. No significant interactions were found between country and demographic factors or comorbidity **(**Fig. 2**)**.

**Fig. 3:**
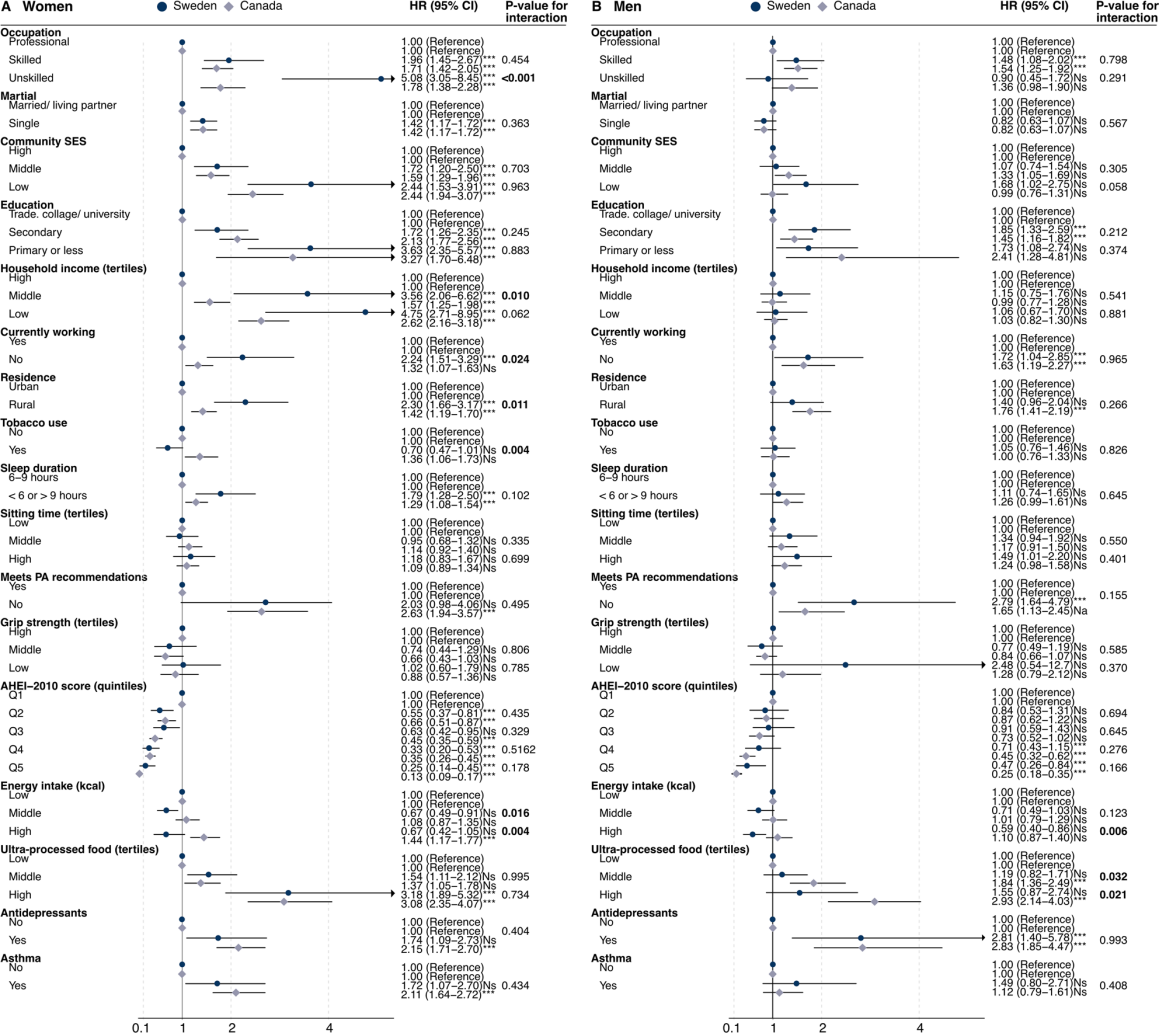
Association between socio-demographic, behavioural risk factors, and comorbidity and odds of having obesity, comparing Sweden with Canada, by (**A**) women and (**B**) men. * are used to indicate interaction between countries and the outcome. *= *p* value: <0.05, **= *p* value: <0.005, ***= *p* value: <0.001. SES= socioeconomic status. Q=quintile. AHEI= Alternative Healthy Eating Index. kcal= kilocalorie. g=gram

In the sex stratified analysis, most factors were similarly associated with obesity as to that of the unstratified analysis. However, it appeared that Swedish, compared with Canadian women, with an unskilled work (OR = 5.08, CI = 3.05–8.45 vs. OR = 1.78, CI = 1.38–2.28, p-value for interaction = < 0.001), middle household income (OR = 3.56, CI = 2.06–6.62 vs. OR = 1.57, CI = 1.25–1.98, p-value for interaction = 0.010), and currently not working (OR = 2.24, CI = 1.51–3.29 vs. OR = 1.32, CI = 1.07–1.63, p-value for interaction = 0.024), had a significantly higher odds of obesity. For men, there were no difference between countries and no increased risk of obesity for these factors. Instead, Canadian men with a middle or high intake of UPF had significantly higher odds of obesity compared with Swedish men (middle: OR = 1.84, CI = 1.36–2.49 vs. OR = 1.19, CI = 0.82–1.71, p-value for interaction = 0.032; high: OR = 2.93, CI = 2.14–4.03 vs. OR = 1.55, CI = 0.87–1.71, p-value for interaction = 0.021), while there was no difference among the women (Fig. 3).

## Discussion

The present study confirms significant differences in the proportion of obesity and obesity related risk factors between participants from Canada and Sweden in a large multinational observational cohort. A substantially higher proportion of the population from Canada (26%) than Sweden 162%) had obesity. Of the 23 selected risk factors for obesity, 19 were identified as important factors in our study. Among these, intake of UPF, AHEI-2010 score, and sex emerged as the most influential factors.

Only a few country-specific behavioural and socioeconomic differences were identified between the two populations that may have contributed to the observed differences in the proportion of obesity. First, high daily intake of UPF was a strongly associated with obesity in both countries, with Canadian men exhibiting particularly higher risk at moderate and high intake levels compared to Swedish men. UPF have been found to substantially contribute to total energy intake in both the United States 35 and Europe 17. In a previous study from the PURE including 138 076 participants from 21 low-, middle-, and high-income countries also found that participants with a high intake of UPF had an increased risk of all-cause mortality, cardiovascular mortality and non-cardiovascular mortality 30.

The daily intake of UPF was substantially higher among Canadians, with men reporting twice the daily intake. The differences in intake could potentially partly be explained by differences in availability of these types of foods between the countries, as previous studies have shown that a high availability in the household [36] and the local environment [13] is associated with an increased risk of obesity. However, as studies focusing on food availability and health outcomes as obesity is limited, in general and in Canada and Sweden, a greater understanding of the food environment and its impact on food consumption and health is needed [37]. Furthermore, as UPF consumption has increased globally over recent decades and now constitutes a substantial proportion of the food supply in many high-income countries [13], the findings of the present study remain highly relevant. Although recent data on UPF intake in Sweden are limited, it is plausible that exposure to and consumption of these products have increased over time. This suggest that Swedish dietary patterns are moving closer to those observed in Canada during the present study period, which could increase the contemporary public health relevance of the associations identified here. Future research should examine more recent trends in UPF consumption and their relation to obesity in different national settings.

Additionally, Canadian participants in the healthiest AHEI-2010 group had significantly lower odds of obesity than their Swedish counterparts in the same group. Notably, Canadians reported substantially higher intake of UPF despite overall better AHEI-2010 scores, a paradox that may reflect differences in how dietary quality and UPF consumption coexist within broader dietary patterns. Further exploration into why these specific dietary factors is more impactful in Canada could inform tailored dietary interventions for different populations.

A second difference identified between countries was in the impact of socioeconomic factors. There was a general trend toward stronger associations between low SES and obesity in Sweden, although only unskilled occupation reached significance. Stratified analysis showed that certain socioeconomic indicators (unskilled occupation, middle income, unemployment, and rural residence) were more strongly associated with obesity among Swedish women compared to Canadian women. These findings suggest that socioeconomic inequalities may be more closely linked to obesity risk among Swedish women than their Canadian counterparts. Furthermore, obesity was associated, regardless of country, with male sex, rural residence, low education, unskilled/skilled occupation, low income, and unemployment. These factors were more common among Canadians, likely contributing to the higher obesity prevalence observed in Canada. While a substantial body of research has established a strong association between SES and unhealthy lifestyle habits, overweight, and obesity [5–7, 38], previous studies have not addressed whether or why low SES may influence individuals from different countries to varying degrees. Some studies, however, support stronger associations between SES and BMI among women than men [39], which aligns with the present findings.

One possible explanation is that the relationship may partly reflect differences in labour market structures [39, 40] and gendered economic inequalities [41], whereby lower BMI in women is more strongly aligned with socially valued standards of physical attractivness and may therefore be associated with socioeconomic advantage [39, 40, 42]. This suggests that the association between SES and obesity among women may not be explained solely by health-related factors, but also by social and structural mechanisms. Future research should aim to uncover the mechanisms underlying these country-specific differences in the relationship between the socioeconomic factors and obesity in women. 

Among men, SES was not significantly associated with obesity. This may partially be explained by the small sample size, particularly in Sweden (*n* = 739 [39%]), which may have limited statistical power and contributed to wider confidence intervals. Thus, sex-stratified findings for men should be interpreted with caution.

Swedish participants reported healthier lifestyle behaviours, such as higher adherence to physical activity recommendations, shorter sitting time, better sleep duration, and lower alcohol consumption, while Canadians had a better diet quality (AHEI-2010 score) and lower tobacco use. However, in the present study, the only behavioural factors, except for dietary factors mentioned above, that were associated with an increased risk of obesity was adherence to physical activity recommendations. Not meeting physical activity recommendations was associated with a marked increased risk of obesity in both countries. These results are in line with previous research, linking physical inactivity to obesity [10]. Overall, a high proportion of individuals in both countries met the physical activity recommendations. More recent data show that physical activity level is declining in both Sweden and Canada, with approximately 73% meeting recommendations in more recent studies [43, 44]. However, due to different population demographics and physical activity assessment between studies, it is difficult to compare activity level.

Among Swedish participants, energy intake showed an inverse relationship with obesity, with decreasing odds of obesity observed as energy intake increased. In contrast, among Canadian participants, a more expected pattern was found, with increasing energy intake associated with higher odds of obesity. These results may be explained by a systematic underestimation of dietary and/or energy intake among Swedish participants with obesity, which have been shown in a previous study using a similar FFQ [44]. Differences in healthcare practices between countries could also play a role. For instance, individuals with obesity in Sweden may have received dietary recommendations to reduce their intake, which may have influenced how they reported their consumption. Additionally, differences in the measurement instruments used to collect dietary data may have contributed to these findings. Systematic errors in portion size estimation—such as varying interpretations of what constitutes a “standard glass” could have affected the accuracy of reported intake. More generally, the Boruta results may have been influenced by how variables were defined and categorized. Thus, although ethnicity, stress, and heavy drinking were not identified as important predictors, this may reflect measurement limitations, categorization thresholds, or limited variability rather than a true absence of association.

## Strengths and limitations

This study has several strengths and limitations. First, the data collection occurred between 2005 and 2009 and may not fully reflect current dietary patterns or health behaviours. However, these data provided a valuable opportunity to compare obesity related factors in two high-income countries using harmonized measures collected within the same time period, thereby strengthening the internal comparability of the cross-country analyses. Although obesity prevalence has continued to increase in both countries since data collection [1], and dietary patterns may also have changed over time, dietary and other health behaviours established earlier in adulthood often remain relatively stable across adult life [45]. In addition, midlife dietary behaviours remain relevant for later-life health, and adherence to healthier dietary patterns such as the AHEI has consistently been associated with more favourable long-term health outcomes [46]. Nevertheless, the primarily as reflecting associations observed during the study period rather than as a direct description of current prevalence levels or dietary behaviours. In particular, the comparatively recent addition of novel medications increasingly used in clinical practice, may have implications for future obesity trends.

Second, although some risk factors were obtained from objective measures (e.g., grip strength, anthropometry), the reliance on self-reported data for lifestyle behaviours, such as physical activity and diet, may introduce reporting biases, and some misclassification is possible. However, the data were used for cross-country comparison and collected using standardized data collection protocols, minimizing the potential for systematic differences between Canada and Sweden.

Furthermore, the use of country-specific, validated, FFQs [26, 27] used by well-trained staff minimized potential errors. Third, although efforts were made to minimize biases in the selection of individuals within each community, the choice of centers within each country was based on feasibility for participating investigators. As a result, the data from PURE may not be fully representative of the entire population of these countries. However, the inclusion of both urban and rural communities ensures substantial diversity in risk factors and contextual variables, increasing the likelihood that PURE findings have broader applicability compared to many previous studies. Furthermore, the underrepresentation of male participants (39%), compared to the original PURE cohort, which included approximately 45% men in high-income countries [25], likely reduced the statistical power of the sex-stratified analyses among men, and thereby limiting the possibility to detect significant associations. Finally, the findings may not be fully generalizable to other populations outside of Canada and Sweden, especially in countries with different healthcare systems, socioeconomic conditions, or cultural norms. Future research could investigate country-specific associations between various risk factors and obesity, offering deeper insights into the unique determinants of obesity within different national contexts, preferably with larger populations. This could help tailor more effective public health strategies and interventions.

## Conclusion

In conclusion, obesity was more prevalent among Canadian participants, particularly men, and several socioeconomic and behavioural factors were found to contribute to this difference. UPF intake was strongly associated with obesity in both countries, with especially pronounced effects among Canadian males. Among women, socioeconomic disadvantage, particularly unemployment and unskilled occupation, was more strongly associated with obesity in Sweden than in Canada, suggesting potential structural or cultural differences in how SES influences health. This study highlights important differences in obesity-related risk factors between Sweden and Canada with UPF consumption, the AHEI-2010 score and socioeconomic disadvantage emerging as key drivers of obesity, with notable sex- and country-specific patterns. These findings underscore the importance of tailored, context-specific public health strategies to address obesity in different national settings, and the need for targeted obesity prevention programs, tailored to the unique needs of each population. Future research should aim to explore the evolving food environments, and investigate the mechanisms behind country-specific associations, particularly among women and disadvantaged groups.

## Supplementary Information

Supplementary Material 1.

## Data Availability

Data from the PURE study are not available for public use. For the PURE study protocol see www.phri.ca/pure.
