# Supplementary material for: Country-specific differences in the association between obesity risk factors in two high-income countries: a cross-sectional analysis from the Prospective Urban Rural Epidemiology (PURE) study
Source: BMC Public Health. 2026 Jun 2;26:1782. doi: 10.1186/s12889-026-27980-3 (PMC13231536; doi:10.1186/s12889-026-27980-3)
Supplement: Supplementary file 1 — Supplementary Material 1. [file 12889_2026_27980_MOESM1_ESM.docx]

**Supplementary appendices: Country specific differences in the association between risk factors for obesity in two high-income countries: the Prospective Urban Rural Epidemiology (PURE) study**

**Supplementary Appendix A: Supplementary Methods:**

**Selection of Countries**

The selection and number of countries included in PURE reflects a balance between involving a diverse range of communities from countries at varying economic levels, encompassing substantial heterogeneity in social and economic conditions and policies, as well as ensuring the feasibility of long-term follow-up by participating centers. As a result, PURE focused on sites where investigators were dedicated to collect high-quality data within the constraints of a low-budget study over a planned 10-year follow-up period, rather than strictly aiming for proportionate global representation.

**Selection of communities**

Within each country, urban and rural communities were chosen based on broad guidelines (see below). Establishing a universally applicable definition of “community” is challenging. In PURE, a community was defied as a group of individuals sharing common characteristics and residing within a specific geographical area. Typically, a large city or large town was not regarded as a single community; rather, communities were selected from different selections of the city – spanning low-, middle-, and high-income areas – and delineated using geographic measures such as a set of contiguous postal codes, a cluster of streets, or a village. In many courtiers, villages served as the primary sampling units for rural areas.

The inclusion of both urban and rural communities was essential, as urban and rural environments in many countries exhibits distinct social and physical characteristics. By sampling both, the study ensured substantial variation in societal factors across PURE communities.

The number of selected communities varied by country, with an emphasis on ensuring social and economic diversity, while considering the local investigators´ ability to sustain follow-up efforts. In Canada, communities from multiple states were included to capture regional diversity in policy, socioeconomic status, culture, and physical environment, while fewer communities were selected in Sweden.

**Selection of communities**

Within each community, the sampling strategy aimed to obtain a broadly representative sample of adults aged 35 to 70 years. The selection of the sampling frame at each center was guided by considerations of both representativeness and feasibility for long-term follow-up, adhering to a broad study guidelines as mentioned above. Once a community was identified, standardized procedures were applied wherever possible for household enumeration, individual identification, recruitment and data collection. For Canada and Sweden, households were initially contacted by mail, followed by a telephone invitation to attend a central clinic. Households were deemed eligible if at least one member was between the ages of 35 and 70 years and intended to reside in their current home for at least four more years.

At least three attempts were made to contact eligible households. All individuals within these households aged 35 to 70 years who provided written informed consent were enrolled in the study. In cases where an eligible household or individual declined participation, demographic information and self-reported data on cardiovascular disease (CVD) risk factors, education, and histories of CVD, cancer and deaths in the household over the past two years were recorded.

To ensure data standardization and high data quality, the study employed a comprehensive operations manual, training workshops, instructional DVDs, regularly communication with study personnel, and standardized reporting forms. Data entery was conducted using a customized database with built-in range and consistency checks, and the data were electronically transferred to the Population Health Research Institute in Hamilton, Ontario, Canada, where further quality checks were implemented.

**Guidelines for Selection of Countries, Communities, Households, and Individuals Recruited to PURE**

| **Countries** |
| --- |
| 1. High-income countries, middle-income countries, and low-income countries, with the bulk of the recruitment from low- and middle-income regions. 2. Committed local investigators with experience in recruiting for population studies. |
| **Communities** |
| 1. Select both urban and rural communities. Use the national definition of the country to determine urban and rural communities. 2. Select rural communities that are isolated (distance of >50 km or lack easy access to commuter transportation) from urban centers. However, consider ability to process bloods samples, e.g., villages in rural developing countries should be within 45-min drive of an appropriate facility. 3. Define community to a geographical area, e.g., using postal codes, catchment area of health service/clinics, census tracts, areas bordered by specific streets or natural borders such as a river bank. 4. Consider feasibility for long-term follow-up, e.g., for urban communities, choose sites that have a stable population such as residential colonies related to specific work sites in developing countries. In rural areas, choose villages that have a stable population. Villages at greater distance from urban centers are less susceptible to large migration to urban centers. 5. Enlist a community organization to facilitate contact with the community, e.g., in urban areas, large employers (government and private), insurance companies, clubs, religious organizations, clinic or hospital service regions. In rural areas, local authorities such as priests or community elders, hospital or clinic, village leader, or local politician. |
| **Individuals** |
| 1. Broadly representative sampling of adults 35 to 70 years within each community unit. 2. Consider feasibility for long-term follow-up when formulating community sampling framework, e.g., small percentage random samples of large communities may be more difficult to follow-up because they are dispersed by distance. In rural areas of developing countries that are not connected by telephone, it may be better to sample entire community (i.e., door-to-door systematic sampling). 3. The method of approach of households/individuals may differ between sites. In MIC and HIC, mail, followed up by phone contact may be the practical first means of contact. In LIC, direct household contact through household visits may be the most appropriate means of first contact. 4. Once recruited, all individuals are invited to a study clinic to complete standardized questionnaires and have a standardized set of measurements. |

**Supplementary Appendix B: Supplementary Tables and Figures:**

**STable 1. Summary of modifiable risk factors.** The risk factors used in the analysis were obtained at the baseline examination which occurred between 2005 to 2009 in Sweden and 2006 to 2009 in Canada, and collected using standardised methods at the community, household, and individual levels (1).

| **Risk factor** | **Definition or method of measurement** | **Category used as a reference in logistic regression models** |
| --- | --- | --- |
| **Anthropometrics** | | |
| High waist/hip ratio | Waist and hip circumference were measured routinely in participants at baseline and used to calculate the waist to hip ratio (WHR). High waist/ hip ratio was defined as > 0.9 for men and > 0.85 for women | - |
| High waist circumference | High waist circumference was defined as ≥102cm for men and ≥88 cm for women. | - |
| **Demographic factors** | | |
| Sex | Self-reported and classified as men or women | Women |
| Area of residence | Urban or rural, as defined above | Urban |
| Community SES | Communities from low-, middle- and high-income areas were selected based on known information of the geographical area | Low |
| **Socioeconomic risk factors** | | |
| Ethnicity | Self-reported. For the present study, ethnicity was categorized as European versus non-European, as the vast majority of participants in both Sweden and Canada self-identified as European and the remaining ethnic groups were too small for meaningful separate analysis. | - |
| Social status | Marred/ living partner | Marred/ living partner |
| Education | Education was self-reported, and classified as low (primary education level or less),  intermediate (secondary school education) or high (college, trade school, or university education) | College, trade school, or university |
| Occupation | Self-reported and classified as 1) professional/ managers, 2) skilled workers and 3) unskilled workers/ homemaker | Professional managers |
| Not working | Self-reported and classified as Currently working yes/ no | Currently working |
| Marital status | Self-reported, and classified as married/ living partner or single | Married/ living partner |
| Household income | Monthly household income and the amount of income spent on food were measured using standardized questionnaires and participant self-report in PURE. To allow for cross-country comparisons, monthly household income was converted to international dollars using country-specific World Bank Purchasing Price Parity (PPP). Household income was divided in tertiles. | Highest tertile |
| Percent of household income spent on food | See household income. Percent of household income spent on food was divided in tertiles. | - |
| **Behavioural factors** | | |
| Tobacco use | Self-reported, currently smoking/ tobacco use, yes (current)/ no (never/former) | No current tobacco use |
| Heavy alcohol consumption | Self-reported alcohol consumption using a standard alcohol consumption frequency  questionnaire. Heavy consumption was classified as drinking one or more drink(s)/ day, yes/ no. For alcohol, we observed that high and former alcohol consumption were both associated with higher risk of mortality, suggesting that in the former group, participants stopped alcohol consumption after suffering the adverse health effects of alcohol. Therefore, both former and high alcohol consumption was calculated as heavy alcohol consumption. | No heavy dinking |
| Global stress | Psychosocial stress was assessed using two single-item questions addressing stress experienced at work and at home. Stress was defined as feeling irritable, anxious, or having difficulty sleeping due to conditions in either setting. For each question, participants were asked to indicate the extent to which they had experienced stress.  Given the high correlation between work-related and home-related stress, a composite global stress scale was constructed as defined in previous studies (2).  The global stress scale was graded as follows:  1. Never experienced stress/ experienced some periods at home or at work, and  2. Experienced several periods at home or at work/ experienced permanent stress at home or at work | - |
| Sleep duration | Self-reported and classified as having a sleep duration of 6-9 hours yes/ no | 6–9 hours |
| Sitting time | Hour, divided in tertiles | Lowest tertile |
| Physical activity | Physical activity was measured using the International Physical Activity Questionnaire and classified as: meeting physical activity guidelines as defined by world health organization (WHO) of at least 150-300 minutes of moderate-intensity or 75-150 minutes of vigorous-intensity aerobic activity weekly (yes/ no). | Meeting recommendations |
| Grip strength | Measured using JAMAR  Dynamometer, kg, dominant hand | Highest tertile of grip strength |
| **Dietary factors**  Diet was measured using country specific, food frequency dietary questionnaires (FFQ). | | |
| Energy intake | Dietary intake was measured using country-specific validated food frequency dietary questionnaires (FFQ). Country specific food composition tables were used to estimate daily energy intake. The daily energy intake was then divided in tertiles. | Lowest tertile |
| AHEI-2010 score | The AHEI-2010 score was based on 10 components: vegetables, fruit, whole grains, nuts and legumes, long chain omega–3 fats, and polyunsaturated fatty acids, sugar-sweetened drinks and fruit juice, alcohol, red and processed meat, and sodium (3). Each component scored from 0 to 10, with a maximum total score of 100, where higher scores indicate healthier diets. The score was divided in quintiles. | Lowest quintile |
| Ultra processed food | Ultra processed food was classified based on the NOVA classification (4). Full definition of ultra processed food in PURE have previously been described (5).  **Canada:** Non-dairy creamer, milk shake, flavoured yogurt, ice cream, fruit drinks, hot chocolate, pizza, French fries, margarine, ham, bacon, hot dog, sausages, luncheon ham, other luncheon meat, packaged breads, cold breakfast cereals, crackers, muffins, crisp snacks, cake, doughnuts, puddings, pies, tarts, cookies, chocolate, candy, gravy, ketchup, sauces, mayonnaise, sugar substitutes, soft drinks, salad dressings  **Sweden:** Ice cream, margarine, pizza, blood puddings, cold cuts, sausages, liver paste, luncheon meat, packaged breads, cold breakfast cereals, crisp bread, French fries, sweet rolls, cookies, biscuits, baked goods, chocolate, candy, sweets, chips, salad dressings, mayonnaise, tomato ketchup, marmalades, soft drinks. The daily intake was divided in tertiles | Lowest tertile |
| **Comorbidity** | | |
| Asthma | Self-reported | No asthma |
| Antidepressant medication | Self-reported | No antidepressant medication |

**SUPPLEMENTARY TABLES AND FIGURES**

**STable 2. Information on missing values**

|  |  |  |  |  |  |
| --- | --- | --- | --- | --- | --- |
|  |  | **Overall**  **(n=5 934)** | **Sweden**  **(n=1 866)** | **Canada**  **(n=4 068)** |  |
|  | **Demographic factors** |  |  |  |  |
|  | Ethnicity | 28 (0.5) | 0 (0.0) | 28 (0.7) |  |
|  | **Socioeconomic factors** |  |  |  |  |
|  | Married/ living partner | 8 (0.1) | 2 (0.1) | 6 (0.1) |  |
|  | Education | 3 (0.0) | 0 (0.0) | 3 (0.1) |  |
|  | Occupation | 16 (0.3) | 1 (0.1) | 15 (0.4) |  |
|  | Household income | 682 (11.5) | 122 (6.5) | 560 (13.8) |  |
|  | Household income, % spent on  food/month | 913 (15.4) | 314 (16.8) | 599 (14.7) |  |
|  | **Behavioural factors** |  |  |  |  |
|  | Tobacco use | 9 (0.2) | 1 (0.1) | 8 (0.2) |  |
|  | Heavy alcohol consumption | 100 (1.7) | 59 (3.2) | 41 (1.0) |  |
|  | Global stress | 66 (1.1) | 18 (1.0) | 48 (1.2) |  |
|  | Sleep duration, 6–9 hours | 21 (0.4) | 6 (0.3) | 15 (0.4) |  |
|  | Meets physical activity  Recommendations | 562 (9.5) | 154 (8.3) | 408 (10.0) |  |
|  | Grip strength, dh | 194 (3.3) | 33 (1.8) | 161 (4.0) |  |
|  | Sitting time, hour | 23 (0.4) | 10 (0.5) | 13 (0.3) |  |
|  | **Comorbidity** |  |  |  |  |
|  | Asthma | 25 (0.4) | 1 (0.1) | 24 (0.6) |  |
|  |  |  |  |  |  |

**STable 3. Baseline characteristics of overweight and obese PURE participants aged 34 to 60 years from Canada and Sweden, with imputation**

|  |  |  | |  | |  |  |
| --- | --- | --- | --- | --- | --- | --- | --- |
|  |  | **Sweden**  **(n=1 866)** | | **Canada**  **(n=4 068)** | | **p-value** |  |
|  |  | Normal weight  (n=1 356) | Obesity  (n=510) | Normal weight  (n=2 362) | Obesity  (n=1 706) |  |  |
|  | **Anthropometrics** |  |  |  |  |  |  |
|  | Weight, kg (SD) | 67.4 (8.8) | 98.0 (13.3) | 63.8 (8.7) | 99.1 (16.4) | <0.001 |  |
|  | Height, cm (SD) | 171.4 (9.1) | 171.4 (9.7) | 167.6 (8.9) | 168.7 (9.7) | <0.001 |  |
|  | BMI, kg/m^2^ (SD) | 22.9 (1.5) | 33.3 (3.5) | 22.6 (1.6) | 34.8 (4.8) | <0.001 |  |
|  | High waist/hip ratio, n (%) * | 325 (24.0) | 408 (80.0) | 650 (27.5) | 1 273 (74.6) | <0.001 |  |
|  | High waist circumference, n (%) ** | 13 (1.0) | 433 (84.9) | 33 (1.4) | 1 446 (84.8) | <0.001 |  |
|  | **Demographic factors** |  |  |  |  |  |  |
|  | Sex, men, n (%) | 481 (35.5) | 258 (50.6) | 746 (31.6) | 834 (48.9) | <0.001 |  |
|  | Age, years (SD) | 48.6 (7.1) | 49.6 (7.0) | 48.4 (6.8) | 50.1 (6.7) | <0.001 |  |
|  | Area of residence, n (%) |  |  |  |  |  |  |
|  | *Rural* | 223 (16.4) | 136 (26.7) | 634 (26.8) | 612 (35.9) | <0.001 |  |
|  | Ethnicity, n (%) |  |  |  |  |  |  |
|  | *European* | 1 290 (95.1) | 477 (93.5) | 2 146 (90.9) | 1 562 (91.6) | <0.001 |  |
|  | Community SES, n (%) |  |  |  |  | <0.001 |  |
|  | *Low* | 170 (12.5) | 95 (18.6) | 558 (23.6) | 486 (28.5) |  |  |
|  | *Middle* | 818 (60.3) | 312 (61.2) | 1 038 (43.9) | 812 (47.6) |  |  |
|  | *High* | 368 (27.1) | 103 (20.2) | 766 (32.4) | 408 (23.9) |  |  |
|  | **Socioeconomic factors** |  |  |  |  |  |  |
|  | Married/ living partner, n (%) | 1 078 (79.5) | 410 (80.4) | 1 886 (79.8) | 1 330 (78.0) | 0.441 |  |
|  | Education, n (%) |  |  |  |  | <0.001 |  |
|  | *Primary or less* | 123 (9.1) | 90 (17.6) | 28 (1.2) | 57 (3.3) |  |  |
|  | *Secondary* | 454 (33.5) | 213 (41.8) | 519 (22.0) | 579 (33.9) |  |  |
|  | *Trade, collage/ university* | 779 (57.4) | 207 (40.6) | 1 815 (76.8) | 1070 (62.7) |  |  |
|  | Occupation, n (%) |  |  |  |  | <0.001 |  |
|  | *Professional* | 720 (53.1) | 195 (38.2) | 1383 (58.6) | 775 (45.4) |  |  |
|  | *Skilled* | 560 (41.3) | 265 (52.0) | 717 (30.4) | 697 (40.9) |  |  |
|  | *Unskilled* | 75 (5.5) | 50 (9.8) | 262 (11.1) | 234 (13.7) |  |  |
|  | Not working, n (%) | 119 (8.8) | 81 (15.9) | 341 (14.4) | 334 (19.6) | <0.001 |  |
|  | Household income, mean (SD) | 5 239.9 (2 704.4) | 4 811.4 (3 065.2) | 5 569.7 (2 009.9) | 4987.27 (2153.3) | <0.001 |  |
|  | Household income, % spent on  food/month, mean (SD) | 11.2 (5.9) | 12.1 (6.3) | 10.8 (6.5) | 11.2 (7.0) | 0.001 |  |
|  | **Behavioural factors** |  |  |  |  |  |  |
|  | Tobacco use, n (%) | 323 (23.8) | 119 (23.3) | 294 (12.4) | 251 (14.7) | <0.001 |  |
|  | Heavy alcohol consumption, n (%) | 215 (15.9) | 92 (18.0) | 664 (28.1) | 447 (26.2) | <0.001 |  |
|  | Global stress, n (%) | 669 (49.3) | 233 (45.7) | 1 051 (44.5) | 852 (49.9) | 0.002 |  |
|  | Sleep duration, 6–9 hours, n (%) | 1 140 (84.1) | 401 (78.6) | 1 743 (73.8) | 1 208 (70.8) | <0.001 |  |
|  | Meets physical activity  recommendations, n (%) | 1 310 (96.6) | 467 (91.6) | 2 245 (95.0) | 1 526 (89.4) | <0.001 |  |
|  | Grip strength, dh, kg (SD) | 37.9 (11.5) | 40.1 (13.8) | 34.1 (11.2) | 36.7 (13.5) | <0.001 |  |
|  | Sitting time, minutes, mean (SD) | 255.5 (128.9) | 274.0 (135.7) | 284.3 (146.8) | 299.1 (152.3) | <0.001 |  |
|  | Dietary intake, mean (SD) |  |  |  |  |  |  |
|  | Energy intake, kcal/ day | 2 120.6 (737.8) | 2 129.5 (776.7) | 2 301.9 (821.9) | 2 470.1 (896.8) | <0.001 |  |
|  | Ultra processed foods, g/ day | 129.5 (129.7) | 166.2 (211.5) | 269.7 (214.0) | 405.8 (319.1) | <0.001 |  |
|  | AHEI score, total*** | 56.4 (10.2) | 52.6 (10.2) | 60.0 (12.7) | 53.3 (11.2) | <0.001 |  |
|  | AHEI components |  |  |  |  |  |  |
|  | *Vegetables, g/ day* | 366.6 (239.2) | 343.5 (227.4) | 486.4 (279.5) | 473.0 (307.9) | <0.001 |  |
|  | *Fruits, g/ day* | 254.9 (189.2) | 239.3 (199.0) | 247.1 (164.1) | 216.6 (164.0) | <0.001 |  |
|  | *Whole grain, g/ day* | 61.8 (55.5) | 55.4 (57.1) | 103.6 (107.0) | 93.1 (97.2) | <0.001 |  |
|  | *Nuts and legumes, g/ day* | 40.8 (47.8) | 36.1 (44.0) | 55.9 (44.4) | 50.7 (42.7) | <0.001 |  |
|  | *OMEGA 3, g/ day* | 9.5 (1.3) | 9.4 (1.6) | 8.5 (2.4) | 8.4 (2.4) | <0.001 |  |
|  | *PUFA, E%* | 4.8 (1.2) | 4.8 (1.2) | 4.7 (1.3) | 4.8 (1.3) | 0.006 |  |
|  | *SSB and fruit juice, g/ day* | 123.2 (155.2) | 135.6 (226.3) | 201.3 (204.7) | 307.3 (308.4) | <0.001 |  |
|  | *Alcohol, g/ day* | 99.6 (124.2) | 93.1 (124.4) | 142.4 (203.7) | 142.3 (242.3) | <0.001 |  |
|  | *Unprocessed read meat*  *and processed meat, g/ day* | 86.7 (46.6) | 102.7 (56.2) | 80.8 (57.8) | 111.6 (65.9) | <0.001 |  |
|  | *Sodium, mg/ day* | 3 342.2 (1 190.6) | 3 490.2 (1 283.5) | 2 880.9 (1 118.5) | 3 203.1 (1 243.3) | <0.001 |  |
|  | **Comorbidity** |  |  |  |  |  |  |
|  | Asthma, n (%) | 89 (6.6) | 51 (10.0) | 199 (8.4) | 219 (12.8) | <0.001 |  |
|  | Antidepressants, n (%) | 78 (5.8) | 51 (10.0) | 205 (8.7) | 273 (16.0) | <0.001 |  |
|  |  |  |  |  |  |  |  |

kg=kilogram. SD=standard deviation. cm=centimetre. BMI= body mass index. m=metre. n=number. SES= socioeconomic status Dh=dominant hand. kcal= kilocalorie. g=gram. AHEI= Alternative Healthy Eating Index. mg=milligram. OMEGA 3=omega–3 fatty acids. PUFA= polyunsaturated fatty acids. SSB=sugar-sweetened beverages.

* High waist/ hip ratio: > 0.9 for men and > 0.85 for women

** High waist circumference: ≥102cm for men and ≥88 cm for women*.*

*** Ranging from 0 to 100, where higher scores indicate healthier diets

**STable 4. Baseline characteristics of cohort, by BMI category and sex, with imputation**

|  |  |  | |  |  |  |  |  |  |  |
| --- | --- | --- | --- | --- | --- | --- | --- | --- | --- | --- |
|  |  | **Sweden**  **(n=1 884)** | |  |  | **Canada**  **(n=4 124)** |  |  |  |  |
|  |  | **Women** |  | **Men** |  | **Women** |  | **Men** |  |  |
|  |  | Normal weight  (n=875) | Obesity  (n=252) | Normal weight  (n=481) | Obesity  (n=258) | Normal weight  (n=1 616) | Obesity  (n=872) | Normal weight  (n=746) | Obesity  (n=834) |  |
|  | **Anthropometrics** |  |  |  |  |  |  |  |  |  |
|  | Weight, kg (SD) | 63.1 (6.2) | 91.3 (11.5) | 75.3 (7.12) | 104.5 (11.6) | 60.1 (6.4) | 93.1 (14.9) | 71.9 (7.5) | 105.3 (15.7) |  |
|  | Height, cm (SD) | 166.7 (6.2) | 164.2 (6.3) | 180.0 (7.1) | 178.4 (6.9) | 163.6 (6.4) | 162.1 (6.4) | 176.3 (7.3) | 175.7 (7.3) |  |
|  | BMI, kg/m^2^ (SD) | 22.7 (1.5) | 33.9 (3.9) | 23.2 (1.3) | 32.8 (2.9) | 22.4 (1.7) | 35.4 (5.1) | 23.1 (1.4) | 34.1 (4.4) |  |
|  | High waist/hip ratio, n (%) * | 122 (13.9) | 163 (64.7) | 203 (42.2) | 245 (95.0) | 333 (20.6) | 501 (57.5) | 317 (42.5) | 772 (92.6) |  |
|  | High waist circumference, n (%) ** | 13 (1.5) | 227 (90.1) | 0 (0.0) | 206 (79.8) | 31 (1.9) | 797 (91.4) | 2 (0.3) | 649 (77.8) |  |
|  | **Demographic factors** |  |  |  |  |  |  |  |  |  |
|  | Sex, men, n (%) | 48.2 (7.0) | 49.5 (7.0) | 49.1 (7.2) | 49.71 (7.01) | 48.4 (6.7) | 50.02 (6.6) | 48.6 (6.8) | 50.2 (6.7) |  |
|  | Age, years (SD) |  |  |  |  |  |  |  |  |  |
|  | Area of residence, n (%) | 140 (16.0) | 77 (30.6) | 83 (17.3) | 59 (22.9) | 450 (27.8) | 308 (35.3) | 184 (24.7) | 304 (36.5) |  |
|  | *Rural* |  |  |  |  |  |  |  |  |  |
|  | Ethnicity, n (%) | 836 (95.5) | 237 (94.0) | 454 (94.4) | 240 (93.0) | 1 477 (91.4) | 792 (90.8) | 669 (89.7) | 770 (92.3) |  |
|  | *European* |  |  |  |  |  |  |  |  |  |
|  | Community SES, n (%) | 110 (12.6) | 48 (19.0) | 60 (12.5) | 47 (18.2) | 359 (22.2) | 294 (33.7) | 199 (26.7) | 192 (23.0) |  |
|  | *Low* | 521 (59.5) | 160 (63.5) | 297 (61.7) | 152 (58.9) | 714 (44.2) | 390 (44.7) | 324 (43.4) | 422 (50.6) |  |
|  | *Middle* | 244 (27.9) | 44 (17.5) | 124 (25.8) | 59 (22.9) | 543 (33.6) | 188 (21.6) | 223 (29.9) | 220 (26.4) |  |
|  | **Socioeconomic factors** |  |  |  |  |  |  |  |  |  |
|  | Married/ living partner, n (%) | 697 (79.7) | 192 (76.2) | 381 (79.2) | 218 (84.5) | 1 276 (79.0) | 624 (71.6) | 610 (81.8) | 706 (84.7) |  |
|  | Education, n (%) |  |  |  |  |  |  |  |  |  |
|  | *Primary or less* | 66 (7.5) | 50 (19.8) | 57 (11.9) | 40 (15.5) | 15 (0.9) | 23 (2.6) | 13 (1.7) | 34 (4.1) |  |
|  | *Secondary* | 297 (33.9) | 101 (40.1) | 157 (32.6) | 112 (43.4) | 344 (21.3) | 321 (36.8) | 175 (23.5) | 258 (30.9) |  |
|  | *Trade, collage/ university* | 512 (58.5) | 101 (40.1) | 267 (55.5) | 106 (41.1) | 1 257 (77.8) | 528 (60.6) | 558 (74.8) | 542 (65.0) |  |
|  | Occupation, n (%) |  |  |  |  |  |  |  |  |  |
|  | *Professional* | 481 (55.0) | 88 (34.9) | 239 (49.7) | 107 (41.5) | 963 (59.6) | 398 (45.6) | 420 (56.3) | 377 (45.2) |  |
|  | *Skilled* | 354 (40.5) | 128 (50.8) | 207 (43.0) | 137 (53.1) | 470 (29.1) | 338 (38.8) | 247 (33.1) | 359 (43.0) |  |
|  | *Unskilled* | 40 (4.6) | 36 (14.3) | 35 (7.3) | 14 (5.4) | 183 (11.3) | 136 (15.6) | 79 (10.6) | 98 (11.8) |  |
|  | Not working, n (%) | 83 (9.5) | 49 (19.4) | 36 (7.5) | 32 (12.4) | 275 (17.0) | 207 (23.7) | 66 (8.8) | 127 (15.2) |  |
|  | Household income, mean (SD) | 5 291.4 (2 771.2) | 4 339.1 (2 883.1) | 5 146.4 (2 578.7) | 5 272.8 (3 171.3) | 5 591.6 (2 020.2) | 4 603.8 (2 169.2) | 5 522.4 (1 987.9) | 5 388.3 (2 062.8) |  |
|  | Household income, % spent on  food/month, mean (SD) | 11.2 (5.9) | 12.6 (6.4) | 11.1 (6.0) | 11.6 (6.3) | 10.8 (6.5) | 11.6 (7.3) | 10.8 (6.4) | 10.7 (6.6) |  |
|  | **Behavioural factors** |  |  |  |  |  |  |  |  |  |
|  | Tobacco use, n (%) | 177 (20.2) | 38 (15.1) | 146 (30.4) | 81 (31.4) | 183 (11.3) | 126 (14.4) | 111 (14.9) | 125 (15.0) |  |
|  | Heavy alcohol consumption, n (%) | 83 (9.5) | 16 (6.3) | 132 (27.4) | 76 (29.5) | 369 (22.8) | 129 (14.8) | 295 (39.5) | 318 (38.1) |  |
|  | Global stress, n (%) | 485 (55.4) | 123 (48.8) | 184 (38.3) | 110 (42.6) | 762 (47.2) | 473 (54.2) | 289 (38.7) | 379 (45.4) |  |
|  | Sleep duration, 6–9 hours, n (%) | 736 (84.1) | 187 (74.2) | 404 (84.0) | 214 (82.9) | 1 141 (70.6) | 567 (65.0) | 602 (80.7) | 641 (76.9) |  |
|  | Meets physical activity  recommendations, n (%) | 853 (97.5) | 240 (95.2) | 457 (95.0) | 227 (88.0) | 1 540 (95.3) | 771 (88.4) | 705 (94.5) | 755 (90.5) |  |
|  | Grip strength, dh, kg (SD) | 31.4 (6.4) | 29.4 (8.3) | 49.7 (9.0) | 50.5 (9.4) | 28.64 (6.7) | 27.1 (7.5) | 45.9 (9.8) | 46.8 (10.6) |  |
|  | Sitting time, minutes, mean (SD) | 256.9 (128.4) | 271.4 (132.2) | 252.92 (129.99) | 276.5 (139.2) | 284.1 (145.0) | 297.2 (152.6) | 284.8 (150.7) | 301.2 (152.1) |  |
|  | Dietary intake, mean (SD) |  |  |  |  |  |  |  |  |  |
|  | Energy intake, kcal/ day | 1 810.2 (496.4) | 1 715.9 (504.2) | 2 685.3 (770.0) | 2 533.5 (784.2) | 2 196.6 (794.3) | 2 372.3 (893.5) | 2 530.0 (835.0) | 2 572.3 (889.3) |  |
|  | Ultra processed foods, g/ day | 111.4 (99.0) | 148.0 (177.7) | 162.6 (167.2) | 184.0 (239.0) | 241.1 (194.9) | 362.5 (299.4) | 331.5 (239.1) | 451.2 (332.6) |  |
|  | AHEI score, total*** | 58.47 (9.5) | 54.45 (10.0) | 52.58 (10.5) | 50.70 (10.0) | 61.4 (11.3) | 54.8 (11.04) | 57.0 (12.1) | 51.73 (11.12) |  |
|  | AHEI components |  |  |  |  |  |  |  |  |  |
|  | *Vegetables, g/ day* | 395.4 (258.0) | 382.8 (242.3) | 314.2 (189.6) | 305.0 (205.1) | 511.7 (292.7) | 511.6 (324.8) | 431.6 (239.7) | 432.6 (283.9) |  |
|  | *Fruits, g/ day* | 288.6 (194.6) | 283.8 (216.9) | 193.6 (162.1) | 195.7 (169.2) | 252.7 (162.1) | 230.9 (168.7) | 235.1 (167.8) | 201.6 (157.6) |  |
|  | *Whole grain, g/ day* | 59.4 (50.7) | 48.5 (47.1) | 66.4 (63.2) | 62.1 (64.8) | 99.9 (102.2) | 90.9 (93.4) | 111.6 (116.5) | 95.5 (101.0) |  |
|  | *Nuts and legumes, g/ day* | 43.5 (53.2) | 35.9 (42.5) | 35.9 (35.4) | 36.4 (45.5) | 57.0 (44.4) | 53.0 (44.8) | 53.5 (44.4) | 48.3 (40.2) |  |
|  | *OMEGA 3, g/ day* | 9.4 (1.4) | 9.2 (1.8) | 9.7 (1.1) | 9.6 (1.2) | 8.4 (2.4) | 8.3 (2.5) | 8.5 (2.4) | 8.5 (2.3) |  |
|  | *PUFA, E%* | 4.9 (1.2) | 4.8 (1.1) | 4.6 (1.0) | 4.8 (1.2) | 4.8 (1.3) | 4.9 (1.3) | 4.5 (1.2) | 4.7 (1.3) |  |
|  | *SSB and fruit juice, g/ day* | 108.7 (137.4) | 119.2 (201.1) | 149.7 (180.4) | 151.6 (247.7) | 173.8 (181.4) | 254.8 (281.6) | 260.9 (237.3) | 362.2 (325.3) |  |
|  | *Alcohol, g/ day* | 74.3 (79.6) | 52.9 (61.7) | 145.7 (169.5) | 132.4 (154.2) | 100.0 (129.1) | 67.9 (145.7) | 234.2 (288.2) | 220.2 (293.4) |  |
|  | *Unprocessed read meat*  *and processed meat, g/ day* | 81.5 (44.1) | 94.8 (54.7) | 96.1 (49.6) | 110.4 (56.7) | 72.5 (51.6) | 97.6 (62.1) | 98.6 (65.9) | 126.2 (66.7) |  |
|  | *Sodium, mg/ day* | 2 875.4 (864.3) | 2 837.6 (921.6) | 4 191.3 (1 233.6) | 4 127.7 (1 267.9) | 2 749.7 (1 080.5) | 3 098.1 (1 238.2) | 3 165.1 (1 147.2) | 3 313.0 (1 234.0) |  |
|  | **Comorbidity** |  |  |  |  |  |  |  |  |  |
|  | Asthma, n (%) | 63 (7.2) | 30 (11.9) | 26 (5.4) | 21 (8.1) | 139 (8.6) | 143 (16.4) | 60 (8.0) | 76 (9.1) |  |
|  | Antidepressants, n (%) | 64 (7.3) | 31 (12.3) | 14 (2.9) | 20 (7.8) | 177 (11.0) | 188 (21.6) | 28 (3.8) | 85 (10.2) |  |
|  |  |  |  |  |  |  |  |  |  |  |

kg=kilogram. SD=standard deviation. cm=centimetre. BMI= body mass index. m=metre. n=number. SES= socioeconomic status Dh=dominant hand. kcal= kilocalorie. g=gram. AHEI= Alternative Healthy Eating Index. mg=milligram. OMEGA 3=omega–3 fatty acids. PUFA= polyunsaturated fatty acids. SSB=sugar-sweetened beverages.

* High waist/ hip ratio: > 0.9 for men and > 0.85 for women

** High waist circumference: ≥102cm for men and ≥88 cm for women*.*

*** Ranging from 0 to 100, where higher scores indicate healthier diets


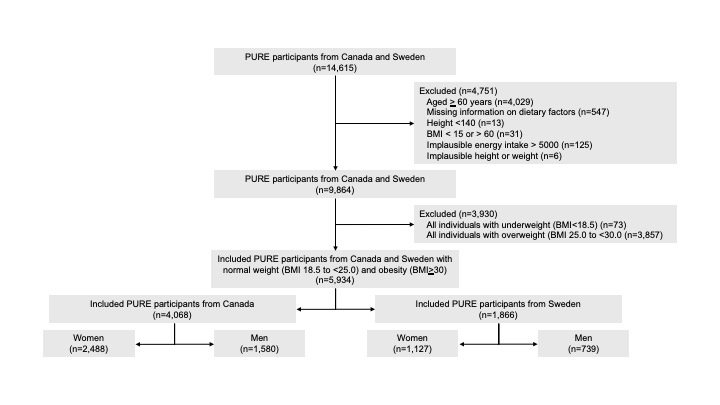
**SFigure 1. Flow chart over inclusion and exclusion of participants**

**
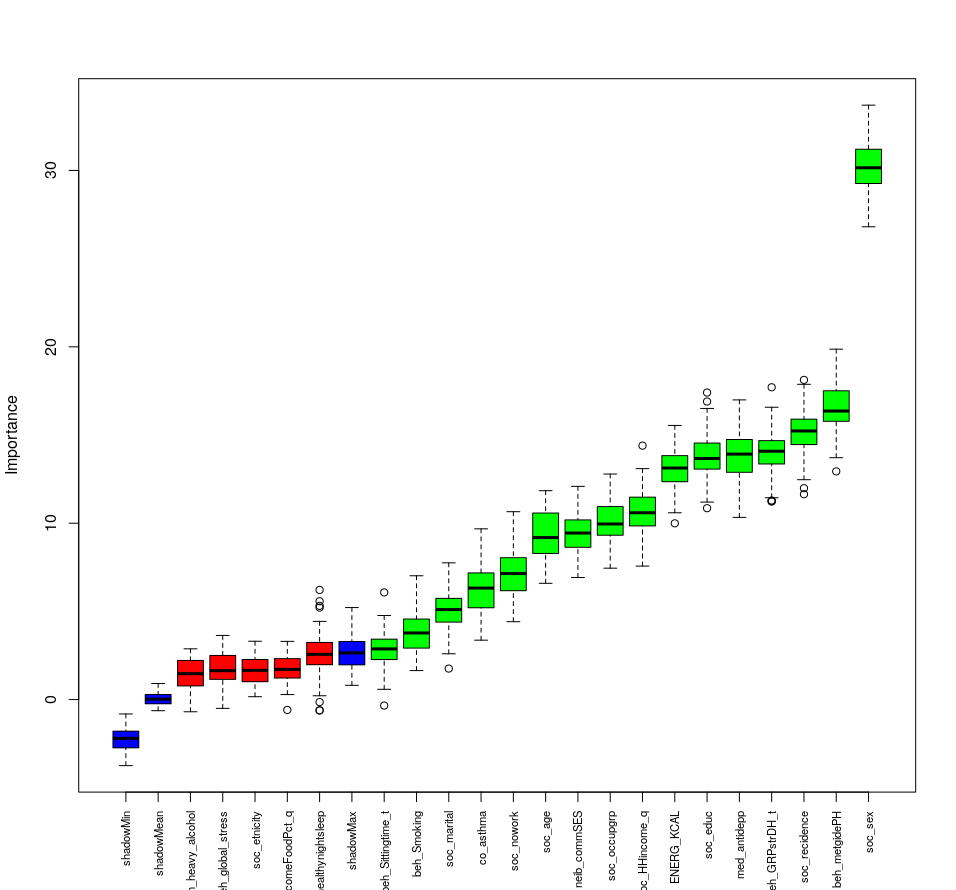
**

**SFigure 2. Sensitivity analysis of variable importance in the Boruta model including only energy intake as the dietary variable, excluding ultra processed food intake and AHEI-2010 score.**


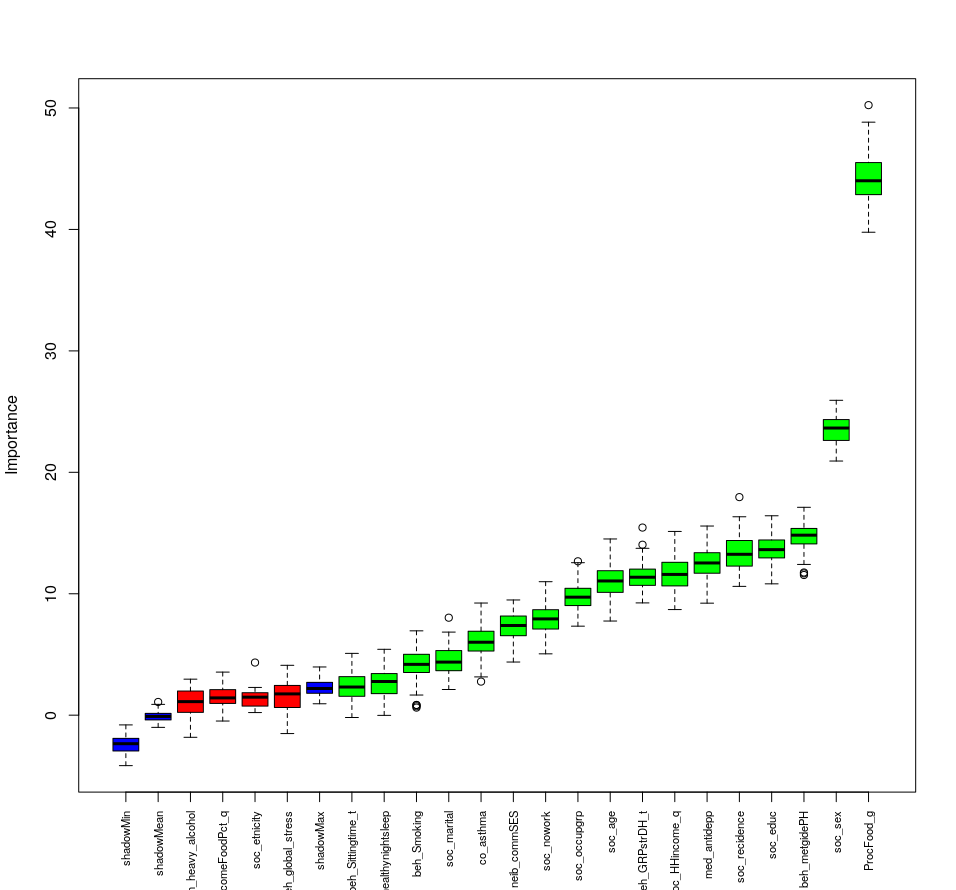


**SFigure 3. Sensitivity analysis of variable importance in the Boruta model including only ultra processed food intake as the dietary variable, excluding energy intake and AHEI-2010 score.**


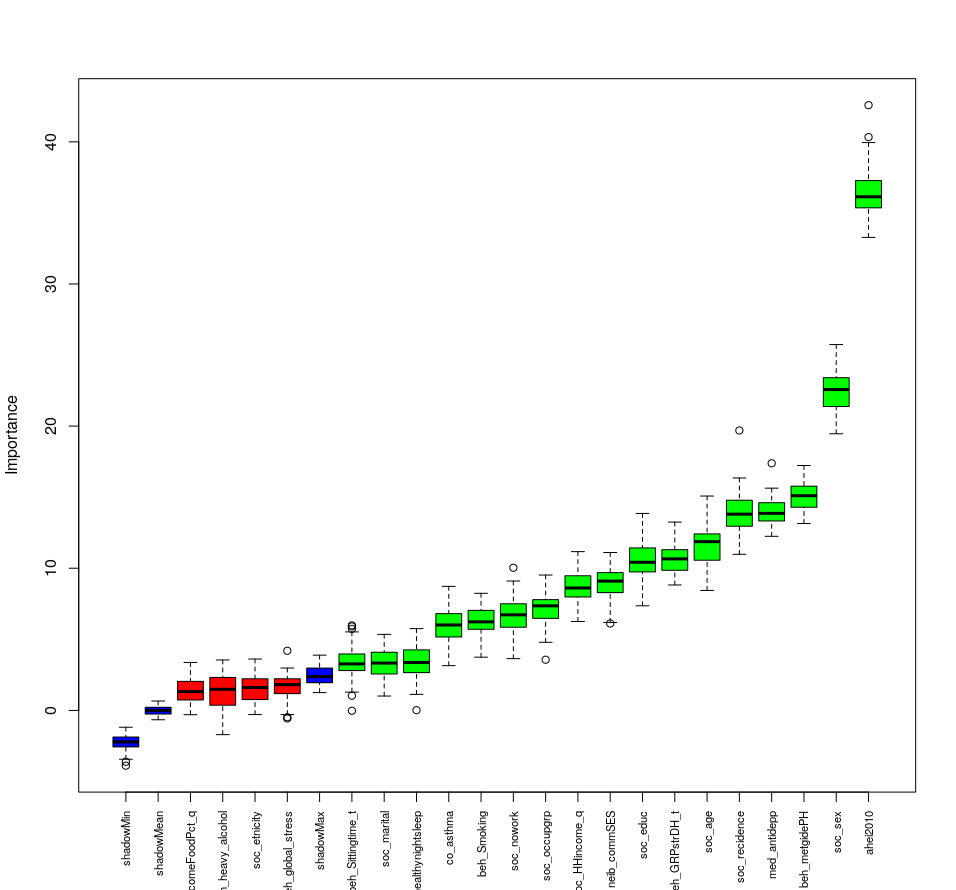


**SFigure 4. Sensitivity analysis of variable importance in the Boruta model including only AHEI-2010 score as the dietary variable, excluding energy intake and ultra processed food intake.**

**References**

1. Teo K, Chow CK, Vaz M, Rangarajan S, Yusuf S. The Prospective Urban Rural Epidemiology (PURE) study: examining the impact of societal influences on chronic noncommunicable diseases in low-, middle-, and high-income countries. Am Heart J. 2009;158(1):1-7.e1.

2. Santosa A, Rosengren A, Ramasundarahettige C, Rangarajan S, Gulec S, Chifamba J, et al. Psychosocial Risk Factors and Cardiovascular Disease and Death in a Population-Based Cohort From 21 Low-, Middle-, and High-Income Countries. JAMA Netw Open. 2021;4(12):e2138920.

3. Chiuve SE, Fung TT, Rimm EB, Hu FB, McCullough ML, Wang M, et al. Alternative dietary indices both strongly predict risk of chronic disease. J Nutr. 2012;142(6):1009-18.

4. Monteiro CA, Cannon G, Moubarac JC, Levy RB, Louzada MLC, Jaime PC. The un Decade of Nutrition, the NOVA food classification and the trouble with ultra-processing. Public health nutrition. 2018;21(1):5-17.

5. Dehghan M, Mente A, Rangarajan S, Mohan V, Swaminathan S, Avezum A, et al. Ultra-processed foods and mortality: analysis from the Prospective Urban and Rural Epidemiology study. The American journal of clinical nutrition. 2023;117(1):55-63.
